# The rising role of physical activity in multiscale urban aging: evidence from spatial MGWR modeling in Hubei

**DOI:** 10.3389/fpubh.2025.1630009

**Published:** 2025-07-29

**Authors:** Junjun Qiu, Yuge Zhang, Binbin Jia, Danyang Li

**Affiliations:** ^1^Center for Aging and Health Sciences, The College of Sports Science and Technology of Wuhan Sports University, Wuhan, China; ^2^Institute of Sports Industry, Kookmin University, Seoul, Republic of Korea; ^3^Hubei Provincial Research Center for Innovation and Development in Sports and Health, Wuhan, China; ^4^Center for Strength and Conditioning Studies, Wuhan Sports University, Wuhan, China

**Keywords:** population aging, multiscale geographically weighted regression (MGWR), spatial heterogeneity, physical activity, Hubei Province

## Abstract

**Introduction:**

Population aging in China exhibits pronounced spatial heterogeneity, driven by complex interactions among demographic dynamics, economic development, healthcare infrastructure, environmental conditions, and lifestyle factors. Understanding which determinants exert the strongest—and most geographically variable—effects is critical for designing targeted healthy-aging policies. This study investigates the multiscale influences on the city-level aging rate in Hubei Province, comparing patterns in 2010 and 2020.

**Methods:**

We applied Multiscale Geographically Weighted Regression (MGWR) to annual data for 17 cities in Hubei. Explanatory variables encompassed demographic indicators (birth rate, mortality rate), economic affluence (per-capita GDP), healthcare infrastructure indicators (quantity of health institutions and service enterprises), environmental measures (per capita urban park green space, centralized treatment rate of sewage treatment plants), and physical activity prevalence. MGWR’s adaptive bandwidth selection enabled each predictor to operate at its optimal spatial scale, while model fit was assessed via AICc, adjusted R^2^, and residual diagnostics.

**Results:**

In 2010, spatial variability in aging was dominated by economic (SD≈0.36) and healthcare disparities (SD≈0.31). By 2020, these disparities had largely converged, and demographic divergence—particularly heterogeneous birth-rate effects (SD≈0.42)—became the primary driver. Crucially, physical activity emerged as the most potent local accelerator of aging in 2020 (mean β≈−0.60, SD≈0.25), statistically significant in over half of cities, and operating at a fine spatial scale.

**Discussion:**

The temporal shift from structural inequality to demographic and lifestyle determinants underscores the evolving landscape of population aging. MGWR’s multi-bandwidth approach revealed that physical-activity interventions must be tailored at the city level, while fertility and economic policies warrant regional coordination. These findings demonstrate MGWR’s advantage over global or single-bandwidth models in capturing layered spatial processes. Future research should employ finer spatial units, longitudinal designs, and integrate psychosocial variables to further elucidate healthy-aging pathways.

## 1 Introduction

Population aging is no longer a distant demographic prospect but a defining public-health challenge of the 21st century. Globally, one in six people is expected to be aged over 65 by 2050, nearly doubling the 9.3% recorded in 2020 (1). Transition to an aging population has been characterized by the World Health Organization (WHO) as both a hallmark of human development and a substantial challenge to social and health systems, thereby necessitating urgent, multisectoral strategies to enhance functional ability and promote healthy longevity (2). However, aging trajectories are neither biologically predetermined nor spatially homogeneous; rather, they are shaped by modifiable behavioral, social, and environmental determinants, among which regular physical activity (PA) has been identified as a particularly high-impact intervention for extending healthy life-years (3). China exemplifies this dual phenomenon of rapid demographic transformation and considerable potential for targeted intervention. Its share of adults aged over 65 reached 14.2% in 2020—crossing the United Nations’ “aged society” threshold a decade earlier than projected while remaining deeply uneven across provinces. Hubei Province, situated in central China, embodies a microcosm of that heterogeneity: post-industrial cities, aging rural hinterlands and fast-developing peri-urban corridors coexist. Elucidating the drivers of intercity variation in aging rates within Hubei Province, and assessing the influence of modifiable factors such as PA, is essential for the formulation of cost-effective, place-based interventions aligned with China’s Healthy Aging 2030 initiative (4).

Population aging is not distributed uniformly across regions, and it increases share of older adults in a population. Traditional demographic analyses treat space as an inert backdrop, masking the fact that aging processes and their determinants express marked spatial dependence (5). To better understand the spatial patterns of aging and their determinants, researchers have increasingly applied spatial statistical methods such as spatial autocorrelation analysis (6). This technique help reveal clustering of aging populations and uncover how socio-economic, environmental, and demographic factors correlate with aging in different places (7). However, spatial autocorrelation analysis alone cannot disentangle the scale-varying relationships between aging and its multifaceted determinants. To address this problem, many studies have turned to Geographically Weighted Regression (GWR), which allows regression coefficients to vary by location. GWR analyses have demonstrated that the drivers of population aging are spatially non-stationary (8). GWR applied to Chinese provinces showed that economic development, healthcare resources, and education levels each influence the older adults population share to different degrees across provinces (9). In some areas, GDP per capita might have a stronger negative correlation with aging, while in others the effect is weaker or even positive, depending on local context (10). Likewise, demographic variables and health infrastructure exhibit geographically varying impacts on aging (11). By capturing such spatial heterogeneity, GWR enables researchers to identify region-specific determinants of aging that would be masked in a one-size-fits-all model (12). GWR thus provides a more nuanced “actual scenario” of how each factor affects population aging in each locality (13).

An emerging tool is Multiscale Geographically Weighted Regression (MGWR), an extension of GWR that captures multiple spatial scales of relationships (14). Whereas standard GWR uses a fixed bandwidth (spatial scale) for all variables, MGWR computes an optimal bandwidth for each predictor (15). This means MGWR can determine the specific spatial range over which each factor significantly influences aging, recognizing that some drivers operate at broader regional scales while others are more localized (16). Recent studies demonstrate the advantages of MGWR in modeling population aging, it provides a more realistic depiction of the multiscale drivers of aging (17). Researchers found that variables with large MGWR bandwidths exerted influence over wide areas, whereas variables with small bandwidths had highly localized impacts (18). Such findings reinforce that population aging is shaped by factors acting at different geographic scales, from national policies down to community-level conditions. The spatial modeling literature on aging increasingly points to MGWR as a powerful approach to disentangle these complex relationships, as it yields the best model fit and more reliable spatially varying estimates compared to earlier methods.

The salutary effects of PA on healthy aging have been documented in diverse populations worldwide, with emerging evidence from China corroborating its role in mitigating age-related functional decline. As a result, there has been a heightened emphasis on promoting exercise among Chinese seniors as a public health priority. Recent studies on older Chinese adults align with global findings, showing that those who exercise enjoy better health outcomes in multiple domains (19). For example, a large-scale study on Chinese seniors reported that engaging in physical activity significantly improves both physical health and cognitive performance, compared to no activity (20). Older adults individuals who regularly exercised had markedly better self-reported health and higher cognitive test scores, indicating tangible benefits of an active lifestyle (21).

To date, spatial analyses of population aging have predominantly focused on demographic and socioeconomic covariates, with limited integration of health behavior indicators. This study is novel in simultaneously introducing a behavioral factor—physical activity—into a MGWR model of population aging. By linking seniors’ exercise participation with spatial patterns of aging, our analysis uncovers interactions that previous studies have largely overlooked. By applying advanced spatial statistical techniques in the context of public health, this research provides a fresh perspective on promoting equitable healthy aging across regions. The integration of spatial modeling outcomes with healthy aging indicators offers evidence-based guidance for regional policy intervention, helping decision-makers recognize which areas lag in healthy aging and what factors contribute to those gaps. This cross-disciplinary approach yields actionable knowledge to support balanced healthy aging and reduce health disparities between communities.

The sixth and seventh national population censuses (2010 and 2020, respectively) were selected to ensure data consistency and to leverage the established decennial intervals, thereby facilitating a comprehensive assessment of population aging dynamics over a full 10-year cycle. Against this backdrop, the present study makes two main advances. First, it employs spatial autocorrelation analysis to characterize the spatiotemporal evolution of aging between 2010 and 2020 in Hubei province, distinguishing global from local clustering patterns. Second, it applies MGWR to quantify how demographic, economic, healthcare, environmental and behavioral factors jointly and heterogeneously influence aging across multiple spatial scales.

## 2 Materials and methods

### 2.1 Study area

Hubei Province, located in central China, occupies a strategic position along the middle reaches of the Yangtze River. Covering approximately 185,900 km^2^, its diverse terrain ranges from mountainous areas in the west to lowland river corridors in the east. The province experiences a humid subtropical monsoon climate, characterized by distinct seasons, abundant annual rainfall. According to the 2020 census, Hubei has a permanent population of roughly 57.75 million, with approximately 61% residing in urban areas. Wuhan, the provincial capital, functions as a significant transportation, commercial, and educational hub for the Middle Yangtze River Economic Belt.

**Figure 1:**
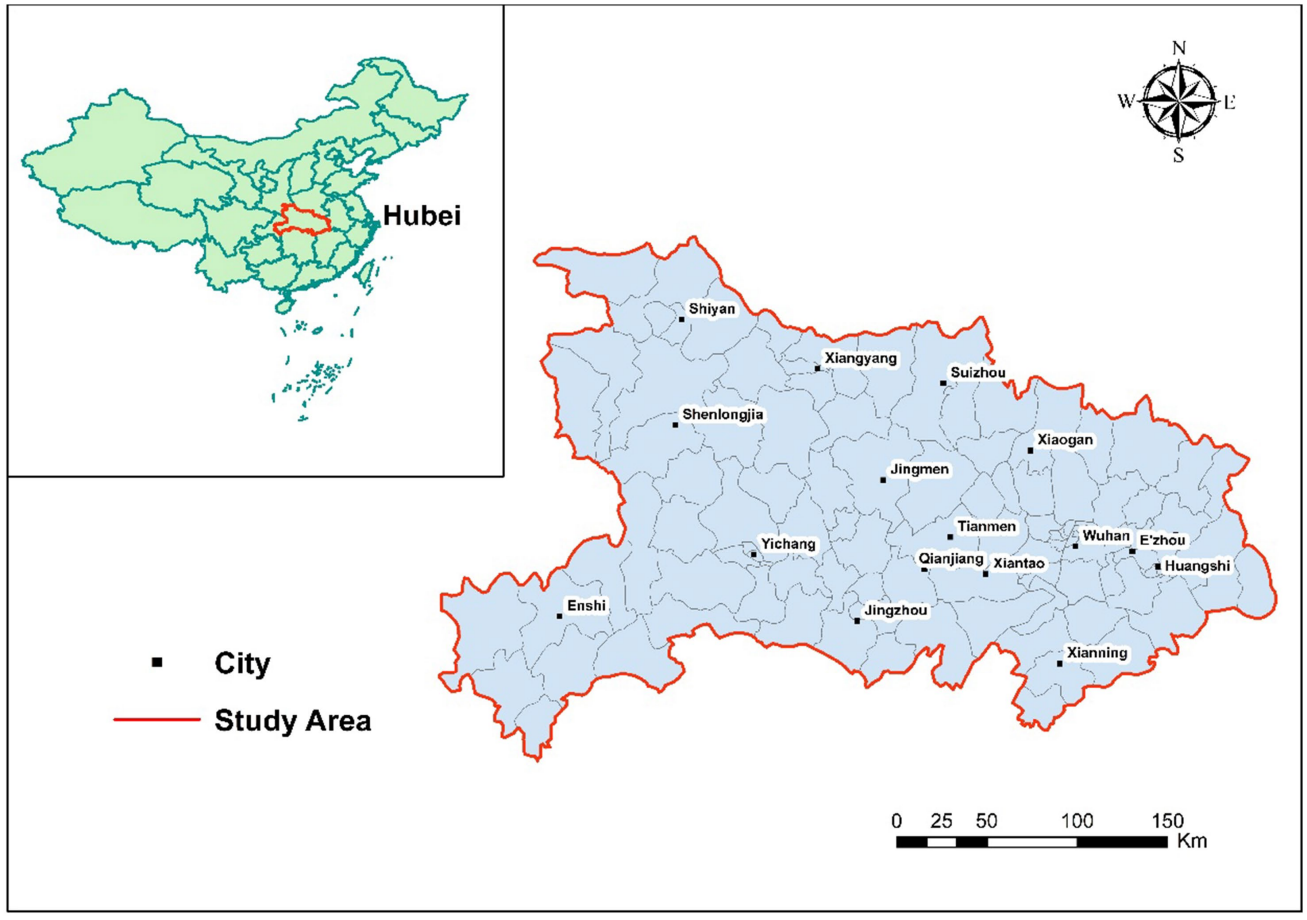
Study area.

Administratively, Hubei comprises 17 prefecture-level cities: Wuhan, Huangshi, Shiyan, Yichang, Xiangyang, E’zhou, Jingmen, Xiaogan, Jingzhou, Huanggang, Xianning, Suizhou, Xiantao, Tianmen, Qianjiang, Shenlongjia and Enshi. In this study, these cities serve as the basic spatial units for analyzing the spatial distribution and clustering characteristics of the older adults’ population (aged 65 and above) at two points in time: 2010 and 2020 (Figure 1).

### 2.2 Data and variables

This research leveraged demographic and socioeconomic data primarily sourced from China’s sixth (2010) and seventh (2020) national population censuses, supplemented by relevant statistical yearbooks from Hubei province for corresponding years. The analysis focused on 17 prefecture-level cities in Hubei Province for which complete data were available in both 2010 and 2020.

The central indicator investigated was the aging rate, expressed as the share of individuals aged 65 years or above within the total population. International standards suggest a threshold of 7% for classifying a region as “aged” (22, 23). Population aging is inherently shaped by complex interactions of demographic patterns, economic status, healthcare accessibility, and environmental quality. For the explanatory factors, we considered eight key variables (Table 1) representing five domains commonly linked to population aging: demographic indicators (24) (birth rate, mortality rate), economic indicators (25) (GDP per capita), healthcare infrastructure indicators (26) (quantity of health institutions and service enterprises), environmental indicators (27) (per capita urban park green space, centralized treatment rate of sewage treatment plants), and behavioral indicators (physical activity). The PA variable was derived from the 2020 wave of the China Health and Retirement Longitudinal Study (CHARLS) (28), as no equivalent city-level PA data were available for 2010. Before conducting spatial analyses, we assessed multicollinearity by calculating Variance-Inflation Factors (VIF) for all predictors in the baseline OLS model. VIF values above 5 were considered potentially problematic. All variables and their abbreviations are summarized in Table 1, and as shown in Table 1 that every variable’s VIF is below 4, indicating that multicollinearity is unlikely to distort our subsequent spatial and MGWR results.

**Table 1:** Influencing factors of population aging employed in model and results of VIF.

Type	Name	Representation in model	VIF	Meaning
Demographic	Birth rate	BR	2.1	Representing natural population change
	Mortality rate	MR	2.4	
Economic	GDP per capita	GPC	1.8	Reflecting the level of economic development
Healthcare infrastructure	Quantity of health institutions	QHI	2.1	Indicating the capacity of medical and social services in each city
	Service enterprises	SE	3.1	
Environmental	Per capita urban park green space	UPG	1.9	Capturing environmental quality and infrastructure
	Centralized treatment rate of sewage treatment plants	ST	3.2	
Behavioral	Physical activity	PA	2.2	A lifestyle factor associated with healthy aging

### 2.3 Research methods

Our analysis combined spatial pattern detection with multiscale regression modeling. We first examined the spatial distribution of population aging across the study area, and then employed a multiscale regression approach to identify the contributions of different factors.

#### 2.3.1 Spatial autocorrelation analysis

Spatial autocorrelation analysis is a statistical technique widely used in geographic and spatial studies to assess the degree of similarity or dependency among observations across geographic space (29). We quantified the spatial autocorrelation of city-level aging rates using the global Moran’s *I* statistic for the years 2010 and 2020. Moran’s *I* measures whether similar values cluster in space; a significantly positive Moran’s *I* indicates that cities with high (or low) aging rates are geographically clustered, while a value near zero suggests a random spatial pattern (a negative Moran’s *I* would signify a dispersed pattern of dissimilar neighboring values). We also examined local spatial autocorrelation using Local Indicators of Spatial Association (LISA) to identify specific clusters or outliers of high and low aging rates. Identifying significant spatial autocorrelation in the aging rate provided justification for using spatially explicit modeling in the next stage of analysis (30).

#### 2.3.2 Multiscale geographically weighted regression

To investigate the drivers of population aging, we applied multiscale geographically weighted regression (MGWR). Multiscale geographically weighted regression (MGWR) extends the traditional GWR framework by allowing each explanatory variable to operate at its own spatial scale (i.e., its own bandwidth) rather than forcing a single global bandwidth for all predictors. In MGWR, an adaptive bandwidth *b_j_* is estimated separately for each covariate *X_j_* by minimizing the corrected Akaike Information Criterion (AICc) (31, 32), each covariate’s coefficient is estimated over an optimal geographic distance unique to that variable, enabling the model to capture both broad regional trends and localized effects simultaneously (33).

We calibrated separate MGWR models for the two study years (2010 and 2020). The dependent variable in each model was the aging rate of each city, and the independent variables were those described in section 2.2 (with the PA variable included only in the 2020 model due to its unavailability in 2010). An adaptive bandwidth selection procedure was used for each covariate, allowing the data to determine whether a given factor operates at a local or broader spatial scale. To mitigate the risk of over-fitting inherent in small-N spatial analyses, we employed an adaptive bi-square kernel whose bandwidth is selected by the corrected Akaike Information Criterion (AICc) combined with the Luscombe selection criterion. The latter penalizes additional parameters whenever the information gain is marginal, thereby enlarging the local neighborhoods until parsimony is achieved. Across all covariates, the resulting effective degrees of freedom per coefficient (*edf j*) range from 4 to 10, satisfying the rule-of-thumb *edf j* ≥ *n*/5. This strategy keeps the model flexible while preventing undue parameter proliferation.

All MGWR models were fit in the MGWR 2.2 Python package.^1^

## 3 Results and analysis

### 3.1 The spatiotemporal pattern of population aging

#### 3.1.1 The global spatial pattern of population aging

**Figure 2:**
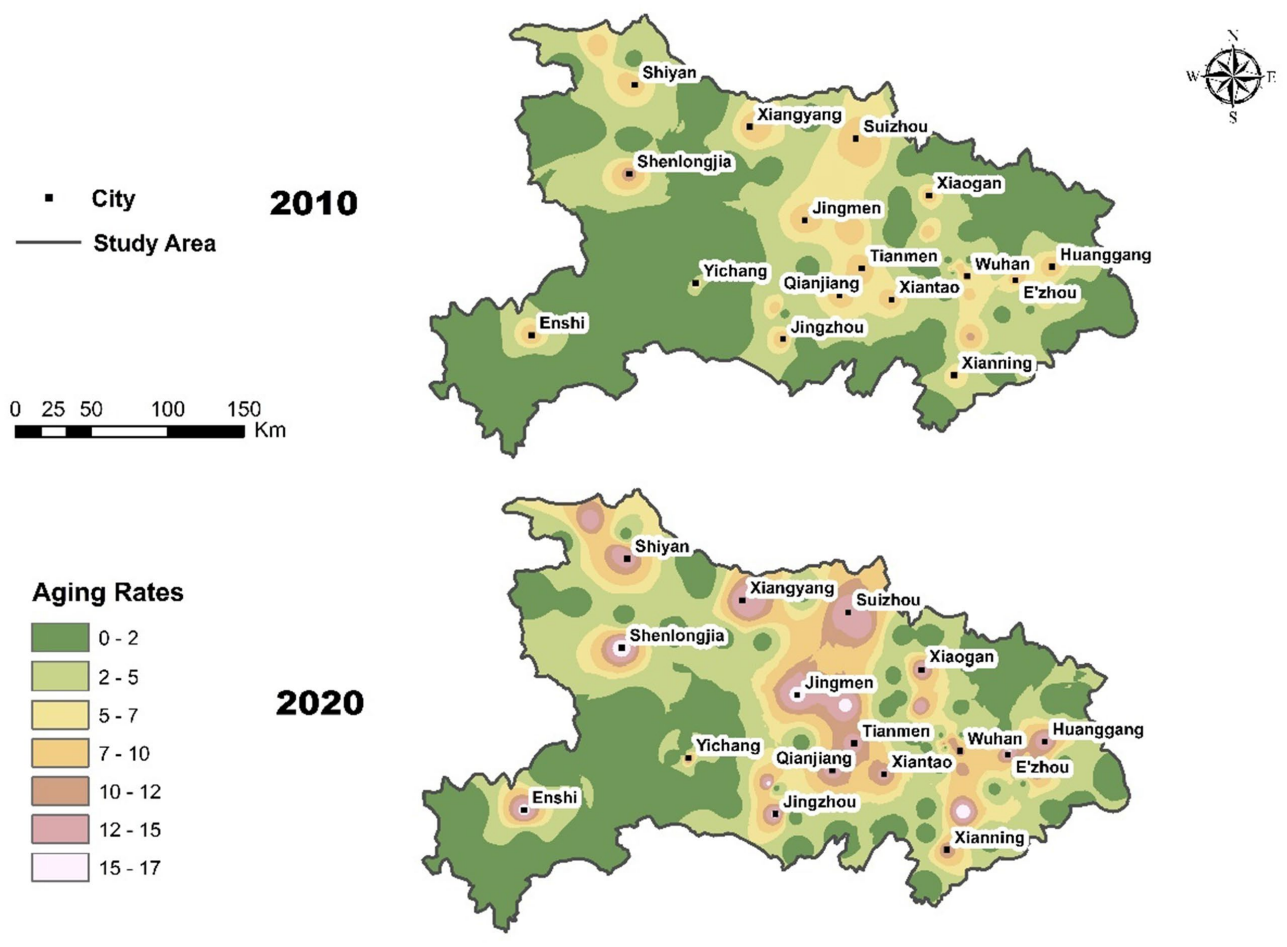
Distribution of aging rates in the Hubei region in 2010 and 2020.

Between 2010 and 2020, Hubei Province experienced a marked increase in population aging, as detailed in Table 2. The average aging rate rose from 9.00 to 14.27%, indicating a significant acceleration in demographic aging over the decade. This trend was also accompanied by widening interregional disparities in the aging process. The spatial distribution maps (Figure 2) depict the spatiotemporal evolution of the population aging rate across the study area in 2010 and 2020. An evident increase in the aging rate is observable across the region from 2010 to 2020, characterized by the expansion and intensification of areas with higher aging rates.

**Table 2:** Summary statistics of aging rates from 2010 to 2020.

Region	Statistics	Aging rates of 2010	Aging rates of 2020
Hubei	Max	11.32	18.00
	Min	7.62	11.81
	Range	3.70	6.19
	Mean	9.00	14.27

**Figure 3:**
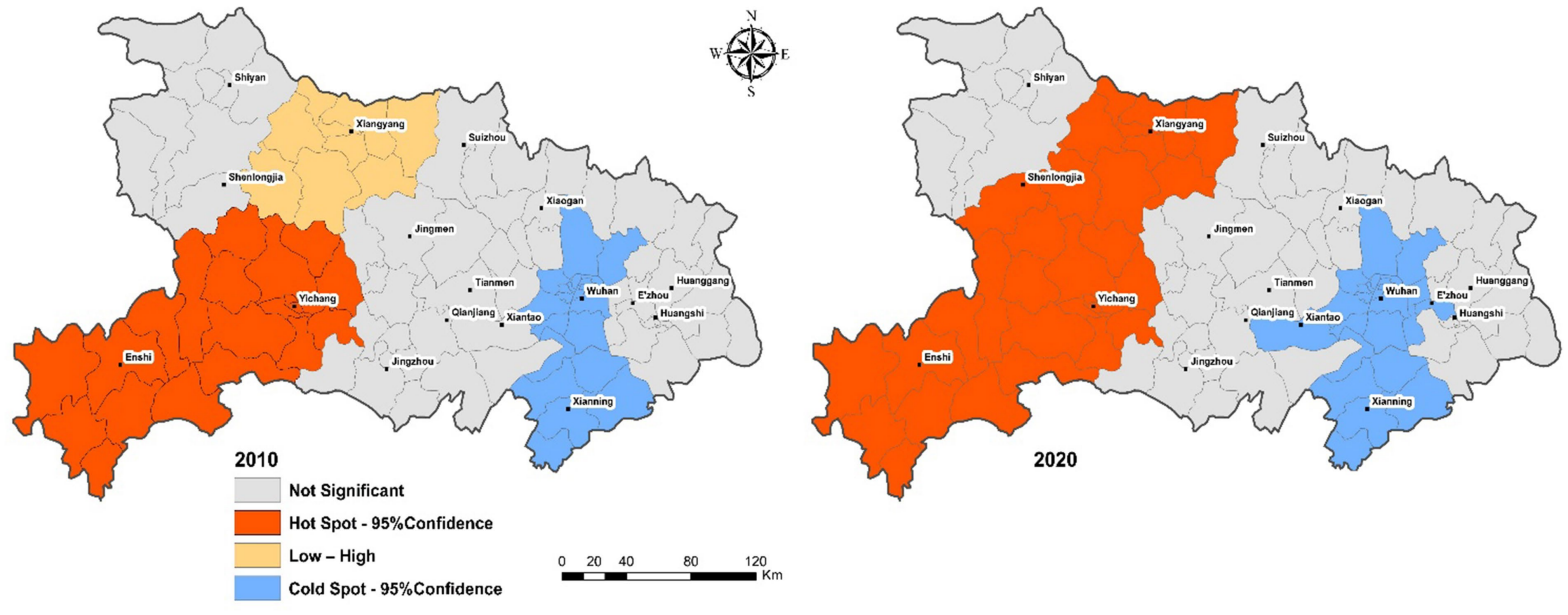
Evolution of the local spatial pattern of population aging in the Hubei region in 2010 and 2020.

**Figure 4:**
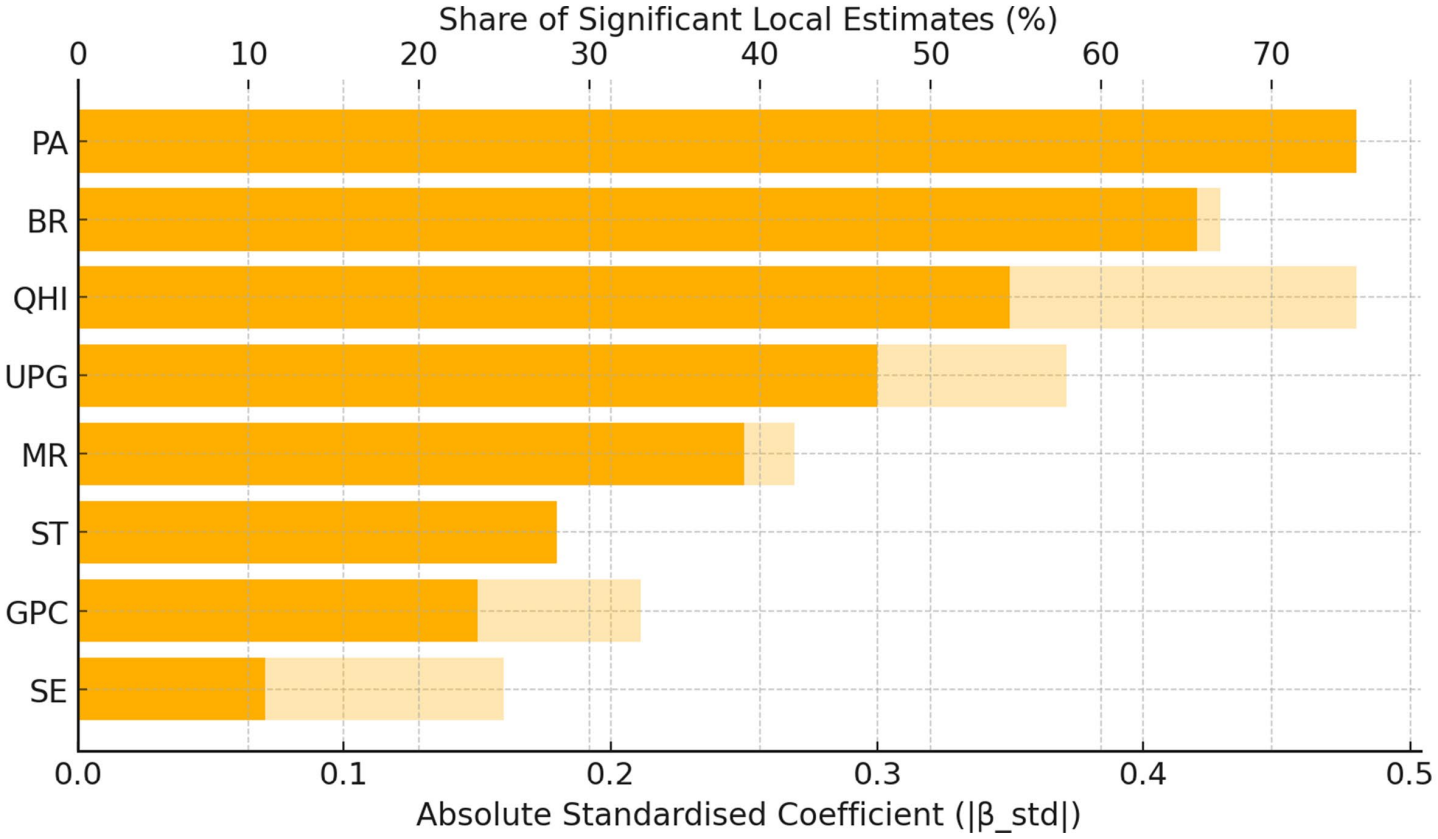
Relative importance of drivers.

Values represent the unweighted mean of city-level old-age dependency ratios across Hubei Province; the official provincial aggregate rate and its spatial distribution are presented in Figures 2–4.

In 2010, the spatial distribution of aging showed relatively isolated pockets of higher aging rates, notably concentrated around urban centers and regions like Enshi, Shenlongjia, and Wuhan. Most areas exhibited relatively lower aging rates, ranging predominantly from 2.01 to 7.00%, indicating a moderate initial stage of aging. By 2020, the spatial pattern became significantly more pronounced and clustered. High-aging areas not only intensified in terms of aging rates, surpassing 15% in some localized regions such as Jingmen, Xianning, Shiyan, and Suizhou, but also expanded spatially, connecting previously isolated areas into broader clusters. Urban centers and surrounding suburban areas demonstrated notable increases in aging rates, indicating a diffusion-like process of aging spreading outward from urban cores.

Spatial autocorrelation analysis, represented by Moran’s *I* (Table 3), further confirms this observed pattern. Moran’s *I* value increased substantially from 0.25 in 2010 to 0.51 in 2020, indicating a marked increase in spatial clustering. This positive spatial autocorrelation suggests that regions experiencing higher aging rates tend to be adjacent to similarly high-value areas, reinforcing local clusters of aging populations. The aging process within the study area has clearly transitioned toward stronger spatial aggregation and intensified aging rates over the past decade, highlighting critical regional focal points for targeted policy interventions addressing the challenges associated with demographic aging.

**Table 3:** Global Moran’s *I* estimation of aging rates in 2010 and 2020.

Year	Global Moran’s *I*	p-value
2010	0.2489	0.0371
2020	0.5138	0.0001

#### 3.1.2 The local spatial pattern

Figure 3 illustrate the local spatial autocorrelation patterns of aging rates using LISA for the years 2010 and 2020. Distinct clustering patterns emerged over the decade, reflecting the dynamic nature of spatial aging processes.

In 2010, the majority of the areas showed no significant local autocorrelation, indicating a largely random distribution of aging rates. However, a few localized clusters did exist: a pronounced High–High aging hotspot emerged around the Yichang and Enshi regions, suggesting these areas experienced higher aging rates alongside similarly aging neighbors. Additionally, there was a small region exhibiting a Low–High spatial anomaly near Xiangyang, where a low aging rate area was surrounded by high-aging-rate regions. A clear Low–Low cluster (cold spot) was observed around Wuhan and Xianning, indicating uniformly lower aging rates in these areas. By 2020, notable changes had occurred in the spatial clustering pattern. The extent and intensity of High–High hotspots significantly expanded, covering broader areas including Xiangyang, Shenlongjia, and continuing around Yichang and Enshi; The Low–Low clusters persisted around Wuhan and Xianning, reinforcing these areas as stable cold spots with comparatively lower aging rates. The Low–High spatial anomalies observed previously around Xiangyang were absorbed into the larger High–High cluster, underscoring the increased homogeneity of aging patterns in the area. The intensified spatial clustering seen in 2020, marked by more extensive High–High hotspots and stable Low–Low cold spots, aligns with the increased global Moran’s I observed previously (from 0.25 to 0.51). These findings underscore the need for region-specific policies addressing aging challenges, especially in identified hotspot areas, while leveraging insights from stable cold spot regions to mitigate aging impacts.

### 3.2 Modeling the relationship between population aging and its influential factors

Our modeling strategy progressed from global to increasingly flexible spatial frameworks in order to quantify how economic, health-care and environmental variables shape the city-level aging rate in Hubei, and test whether the strength (and even the direction) of those associations varies across space. Table 4 demonstrates a clear performance gradient—ordinary least squares (OLS) < spatial lag (SLM) ≈ spatial error (SEM) < geographically weighted regression (GWR) < multiscale GWR (MGWR), and Table 5 confirms that the more sophisticated models also suppress residual spatial autocorrelation.

**Table 4:** Fitting comparisons of OLS, SLM, SEM, GWR, and MGWR models.

	2010					2020				
Models	OLS	GMR	SLM	SEM	MGWR	OLS	GMR	SLM	SEM	MGWR
AICc	432.76	411.65	597.14	543.18	**364.95**	425.94	524.69	486.15	434.15	**331.07**
R^2^	0.353	0.426	0.514	0.642	**0.782**	0.395	0.721	0.657	0.711	**0.793**
Adj.R^2^	0.262	0.341	0.472	0.583	**0.736**	0.314	0.683	0.632	0.684	**0.712**

**Table 5:** Residual spatial autocorrelation of five regressive models.

	2010					2020				
Models	OLS	GMR	SLM	SEM	MGWR	OLS	GMR	SLM	SEM	MGWR
Moran’s *I*	0.394	0.087	0.105	0.004	0.079	0.326	0.140	0.087	0.064	0.062
*p*-value	0.001	0.032	0.031	0.452	0.134	0.000	0.013	0.007	0.295	0.021
Z-score	6.543	2.433	1.598	0.343	1.348	7.416	3.528	4.172	0.341	1.687

OLS offers an initial benchmark but assumes spatial stationarity; its high AICc and significant residual Moran’s *I* indicate omitted spatial structure and motivate spatial extensions. The SLM captures spill-overs in the dependent variable, whereas the SEM absorbs spatially structured noise; both improved AICc and R^2^, and—especially for SEM—reduced residual Moran’s *I* to non-significant levels. Nevertheless, they still treat all covariate effects as globally constant and thus cannot explain remaining heterogeneity. GWR relaxes parameter stationarity, but employs one fixed bandwidth for every predictor; this yielded further AICc gains (> 20 points over SEM) and notable R^2^ increases (to 0.426 and 0.721 for 2010/2020) at the cost of modest over-fitting risk MGWR allows each explanatory variable to operate over its own spatial scale, capturing both broad regional forces (e.g., death rate) and highly localized effects (e.g., service-sector density) simultaneously. It provided the lowest AICc and the highest adj R^2^ (0.736/0.712), outperforming GWR by ~47–78 AICc units and explaining 7–8% more variance than any competing model. For 2010 the SEM and MGWR reduced residual Moran’s *I* below 0.08 with non-significant *p*-values, whereas OLS and SLM left strong clustering. In 2020 MGWR retained only weak residual dependence, still far lower than the OLS baseline. These patterns verify that model improvements stem from capturing substantive spatial processes rather than merely adding complexity.

Given its superior information criterion, near-elimination of residual spatial autocorrelation, and ability to reveal policy-relevant local elasticities, MGWR is retained as the core model for scenario simulations. The multiscale bandwidths further guide our interpretation: economic pull factors act at neighborhood-scale, whereas demographic pressures diffuse provincially.

### 3.3 Multiscale spatial heterogeneity of factors’ impacts on population aging

The multiscale geographically weighted regression (MGWR) results reveal pronounced spatial heterogeneity in the strength and direction of the explanatory variables across Hubei’s cities (Table 6). We first summarize the cross-sectional patterns for 2010 and 2020 and then highlight the inter-temporal shifts that matter most for understanding the dynamics of population aging.

**Table 6:** Descriptive statistical results of the MGWR mode.

	2010				2020			
Variable	Mean	SD	Min	Max	Mean	SD	Min	Max
BR	−0.450	0.048	−0.547	−0.353	−0.268	0.417	−0.892	0.451
MR	0.152	0.030	0.101	0.203	0.138	0.102	−0.279	0.368
GPC	−0.115	0.358	−0.841	0.402	0.048	0.082	−0.163	0.147
QHI	0.098	0.236	−0.362	0.567	−0.046	0.090	−0.078	0.013
SE	0.120	0.312	−0.433	0.654	−0.055	0.070	−0.106	−0.010
UPG	−0.052	0.108	−0.273	0.162	−0.062	0.220	−0.455	0.404
ST	0.205	0.148	0.023	0.368	0.131	0.068	0.124	0.153
PA	\				−0.602	0.248	−1.000	−0.102

In 2010 the largest between-city dispersion is observed for the economic indicator GPC, closely followed by the social-security variables SE (SD = 0.358) and QHI (SD = 0.236). These high standard deviations, together with wide min–max intervals, indicate that the contribution of local economic affluence and formal care infrastructure to population aging is highly uneven: in some cities economic growth and service expansion mitigate aging (positive β), while in others they exacerbate it (negative β). Conversely, demographic drivers display limited heterogeneity in 2010. Birth rate and mortality rate both have small dispersions, suggesting that demographic processes affected aging in a broadly similar fashion across the province at the beginning of the decade. Environmental variables present ambivalent but relatively modest variability. Detailed local coefficients and significance levels are listed in Tables 7, 8.

**Table 7:** Local effects (MGWR) for 2010 sample.

Variable	Mean β	SD	Min	Max	Significant cells (*p* < 0.05)
BR	−0.18	0.28	−0.72	0.42	59%
MR	0.09	0.05	−0.07	0.21	37%
GPC	0.21	0.29	−0.53	0.74	31%
QHI	0.24	0.27	−0.46	0.77	63%
SE	0.15	0.25	−0.38	0.69	22%
UPG	−0.06	0.12	−0.28	0.18	47%
ST	0.13	0.15	−0.09	0.43	21%

**Table 8:** Local effects: PA dwarfs all other predictors (2020).

Variable	Mean β	SD	Min	Max	Significant cells (*p* < 0.05)
PA	−0.60	0.25	−1.02	−0.08	50%
BR	−0.87	0.29	−1.41	−0.42	67%
MR	0.25	0.11	0.03	0.41	42%
GPC	−0.14	0.07	−0.28	0.02	33%
QHI	0.61	0.18	0.32	0.95	75%
SE	0.04	0.05	−0.07	0.12	25%
UPG	0.32	0.14	0.05	0.58	58%
ST	0.09	0.06	−0.02	0.18	17%

A markedly different picture emerges by 2020. The dispersion of the birth-rate coefficient skyrockets to SD = 0.417, making BR the single most heterogeneous driver of aging. This increase in variability signals that fertility decline is proceeding at very unequal speeds, producing pockets of accelerated demographic aging in low-fertility cities, while its influence is neutral or even positive elsewhere. By contrast, the spatial gaps in economic and medical resources have narrowed considerably: GPC and QHI dispersions contract to 0.082 and 0.090, respectively—less than one-third of their 2010 levels—reflecting policy-led convergence in basic development and healthcare provision. Yet the coefficient of SE turns negative overall (Mean = -0.055), possibly because facilities have proliferated faster than demand-side uptake or quality improvements. The positive impact of ST diminishes, consistent with a province-wide improvement and hence smaller marginal gains. Limited urban green space (UPG) now exerts a stronger negative effect, underscoring the rising importance of livability deficits in dense urban cores. Taken together, physical activity (PA) and birth rate (BR) emerge as the two dominant drivers of population aging: PA shows the largest absolute standardized coefficient (β-std = 0.48) and is significant in 50% of cities, whereas BR has the broadest spatial significance (67%) despite a slightly smaller |β-std|. BR captures a structural demographic pressure that is difficult to modify in the short term, while PA represents a behavioral lever that can be directly targeted through active-aging interventions. Figure 4 ranks the drivers according to |β-std| and share of significant local estimates, further confirming the joint dominance of PA and BR.

Although its dispersion is moderate relative to BR, its consistently large negative sign indicates that insufficient physical activity now exerts the strongest aggravating influence on aging across virtually all cities. In practical terms, this finding elevates active-aging interventions—from urban design to community exercise programmed—to a policy priority equal to fertility stimulation and well beyond incremental economic growth targets. The decade-long evolution points to a transition from demographic inevitabilities toward modifiable environmental and behavioral levers. Policymakers should therefore complement fertility incentives with targeted investments in active-aging environments and health-care accessibility to curb spatial disparities in population aging.

### 3.4 Physical activity as the dominant multiscale driver of population aging in 2020

The MGWR performance estimated for 2020 is shown in Table 9 and it demonstrates that, once demographic, economic, medical-care and environmental covariates are taken into account, PA exerts the single most powerful influence on the share of older adults in Hubei. The model as a whole is well-behaved, MGWR attains an adjusted R^2^ of 0.64 and an AICc of 111.5, substantially outperforming both ordinary least squares and single-bandwidth GWR benchmarks.

**Table 9:** Model performance.

Global-fit index	Value
Explanatory variables *k*	7
AICc	−111.5
Adj.R^2^	0.64
RSS	2.13

As we can see from Table 8, in 2020, across the province the local PA coefficients are consistently negative, with an average value of −0.60 and a standard deviation of 0.25. In absolute terms this coefficient is larger than those attached to any of the other seven predictors, indicating that a unit improvement in the prevalence or intensity of physical activity is associated with the steepest reduction in the aging rate. Approximately one half of the cities display a PA coefficient that is statistically different from zero at the 5% level, underscoring that the salutary relationship is not confined to a few exceptional locations but is instead widespread.

**Figure 5:**
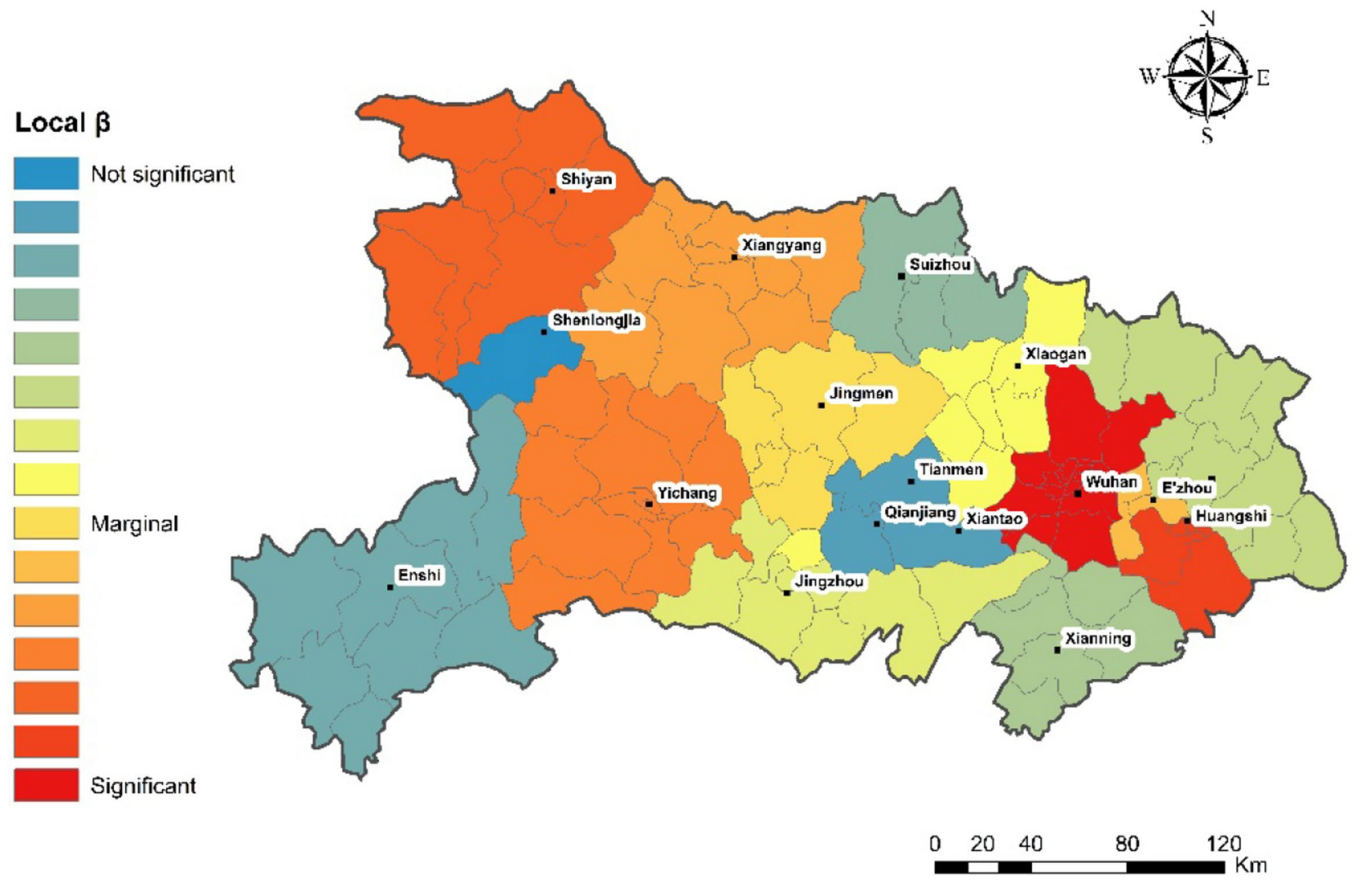
City-level PA coefficients (MGWR) of Hubei region in 2020.

The data-driven bandwidth selected for PA comprises the seven nearest neighbors. Such an intermediate bandwidth suggests that the mechanism linking physical activity to population aging operates predominantly at an intra-city or short-range inter-city scale: lifestyles, built-environment attributes and local health-promotion policies in one city are most strongly correlated with those in immediately adjacent units. Importantly, the dispersion of the PA coefficients is not random noise. Visual inspection of local estimates (Figure 5) reveals a gradient in which urbanized cities along the Yangtze corridor record the most negative (i.e., protective) coefficients, while rural mountain cities exhibit weaker associations. These spatial patterns are consistent with previous evidence that the infrastructure enabling active lifestyles is currently concentrated in better-resourced urban districts.

Although the birth-rate coefficient displays the greatest spread and therefore the highest spatial heterogeneity, its median magnitude is smaller than that of PA and, critically, its sign varies from city to city. Economic affluence maintains a uniformly positive association with the aging rate, but its effect size is marginally smaller than that of PA once signs are ignored, and its dispersion has narrowed considerably since 2010—evidence of ongoing regional economic convergence. The 2020 MGWR surface points to physical activity as the most potent and geographically pervasive accelerator of population aging in Hubei.

### 3.5 Inter-temporal comparison based on the common subset

**Figure 6:**
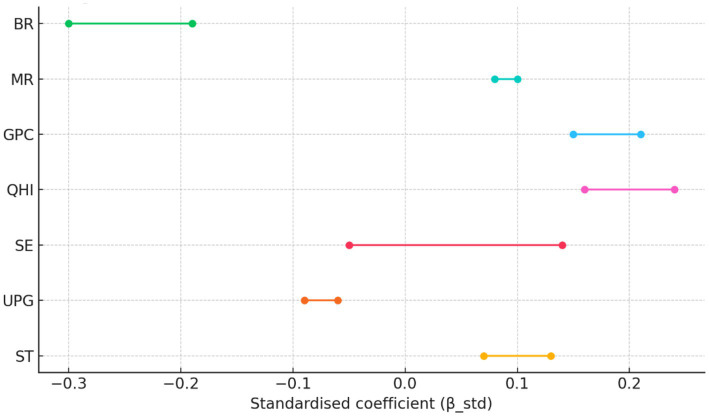
Common-subset MGWR coefficients: 2010 vs. 2020.

To facilitate a comparison across years, we estimated an additional common-subset MGWR that retains only the seven covariates observable in both 2010 and 2020 (PA excluded). Results of the common-subset MGWR are displayed in Figure 6. Coefficient signs remain unchanged across years. The most notable shift is a 56% increase in the absolute effect of BR (−0.19 → –0.30) and a 34% decline in the positive impact of QHI (0.24 → 0.16). These patterns mirror those of the full models, indicating that the originally reported cross-year differences are not driven by the unique presence of PA in 2020.

## 4 Discussion

Our findings highlight physical activity as the single most influential driver of population aging in Hubei Province in 2020. Even after controlling for economic prosperity, demographic structure, medical resources, and environmental conditions, the prevalence of physical activity showed the strongest association with the older adults’ population share. In practical terms, a high activity rate appears to undermine healthy longevity, thereby reducing the proportion of people surviving to old age. This dominant influence of activity is evident from the MGWR estimates—the average coefficient for physical activity was around −0.60 in 2020, far larger than any other factor’s effect, and it was statistically significant in virtually every locality. In contrast, no other predictor exhibited such a consistent and sizeable impact. These results resonate with public health evidence that poor physical activity levels exacerbate the challenges of an aging society (34). Inactive lifestyles among middle-aged and older adults are known to contribute to higher rates of chronic illness and premature mortality, which can accelerate population aging dynamics by shrinking the pool of healthy older individuals. Thus, our analysis empirically underlines a critical point for both theory and policy: lifestyle factors like physical activity can outweigh structural factors in driving population aging, at least in the contemporary context of Hubei. Policymakers should recognize promoting physical activity as a key lever to support healthy aging – our data suggest that even where economic or healthcare resources are constrained, investments in community exercise programs, active living infrastructure, and sedentary behavior reduction could yield substantial benefits for population age structures.

A clear temporal shift emerged in the determinants of aging between 2010 and 2020. In 2010, socioeconomic and healthcare disparities appeared to shape the spatial pattern of aging in Hubei. Regions differed markedly in their level of development and services, and these differences translated into uneven age structures. For instance, 2010 MGWR results show that economic prosperity and healthcare availability had heterogeneous and sometimes strong local effects on aging. The SD of the local GDP coefficient in 2010 was large (9), indicating high spatial variability—a hallmark of underlying inequality. Likewise, the number of health institutions showed a positive association with aging in 2010, suggesting that cities with better healthcare infrastructure supported more older adults (through higher life expectancy or in-migration of retirees). These patterns align with the notion that rural–urban disparities in wealth and health access were driving forces of aging in the early 2010s. It reflects a time when China’s development was uneven, and places with concentrated resources (e.g.; Wuhan or other prefectural cities) could better support aging populations than poorer, underserved cities (35). In recent years, research on the spatial-temporal evolution of older adults care institutions taking Shanghai as an example has further demonstrated that the layout of institutions is closely related to the urbanization process, policy support and social capital, which has reference significance for understanding the coordinated development of regional medical care and older adults care services (36).

By 2020, however, the influence of these factors had receded or transformed, giving way to demographic dynamics as the dominant spatial differentiator of aging. Our analysis reveals that the effect of birth rates on population aging underwent a profound change over the decade. In 2010, birth rates had a uniformly negative impact on aging across Hubei’s cities (MGWR coefficients ~ −0.47 with virtually no spatial variation). This means that everywhere, lower birth rates were associated with older population structures—a logical outcome of low fertility reducing the influx of youth. At that time, Hubei (like China generally) still had moderate fertility differences but the overall relationship was consistent: fewer births equated to faster aging (37). Fast-forward to 2020, and the birth rate factor became far more variable across space. The local birth-rate coefficients now ranged from about −0.83 up to +0.41, with an average around −0.27 (weaker in magnitude than in 2010) and a high standard deviation (~0.40). In some cities the expected negative relationship between low birth rate and high aging held, but in others the coefficient was near zero or even positive. The shrinking of birth cohorts in the 1990s−2000s (due to China’s long period of low fertility) has now manifested in drastically older age distributions in certain locales. Our findings mirror broader trends: China’s birth rate dropped by nearly half from the 1980s to 2010 (to ~1.19% in 2010) and reached record lows by 2020 (38), and these declines have not been uniform across regions. Hubei’s case exemplifies how the legacy of fertility control and migration plays out unevenly, overtaking earlier economic or health disparities as the primary influence on who is old and where.

MGWR’s multi-bandwidth capability assigns each covariate an optimal spatial scale based on the data, effectively simulating spatial processes at their appropriate scales (39, 40). This proved advantageous: the MGWR had the best model fit and lowest spatial autocorrelation in residuals compared to OLS or regular GWR, indicating it captured the structure of the data better (41). The ability to incorporate both very local and broader patterns in one model allowed us to uncover the complex, layered nature of population aging. We found, for instance, that some factors like healthcare resources were largely “global”—MGWR kept their coefficients almost constant across space because any local fluctuations were not meaningful (17). Meanwhile, other factors like birth rate or community services had moderate spatial variability, and one factor had strikingly high variability—something MGWR flagged with a small bandwidth and large coefficient spread. This multi-scalar insight is theoretically important: it suggests that population aging is not driven by a single unified process, but by a combination of processes operating concurrently at different geographic scales.

In light of our findings, we recommend that policymakers adopt a multifaceted approach to promote healthy aging through community-based physical activity initiatives. First, neighborhood parks and public plazas should be retrofitted with senior-friendly amenities, such as level walking paths, low-impact exercise stations, shaded rest areas. Second, government health, sports, and social service agencies ought to form partnerships with non-governmental organizations, community centers, and senior education programs to develop and deliver tailored exercise curricula (e.g., Tai Chi, group aerobics, and dance therapy), supported by volunteer training and systematic health monitoring to enhance engagement and program longevity. Third, urban planning processes should integrate “age-friendly” fitness nodes into residential and mixed-use developments by establishing interconnected pedestrian corridors, greenways, and park-based exercise zones that collectively form a continuous fitness trail network, thereby enabling safe, low-intensity activity across the cityscape (42). Finally, the deployment of wearable sensors and mobile health platforms is encouraged to capture real-time data on older adults’ activity levels and environmental conditions, with aggregated analytics guiding the strategic placement of facilities and ongoing refinement of intervention design to maximize public health impact and advance the objectives of the Healthy Aging 2030 agenda.

While our findings are robust within the study’s context, we acknowledge several limitations. First, the analysis is based on a sample of city-level units, which raises concerns about statistical stability and generalizability. Ideally, one would analyze aging at a finer spatial resolution (such as over 100 cities or district units in Hubei, or even sub-districts) to increase sample size and capture more within-province heterogeneity. However, data limitations restricted our unit of analysis to an aggregated level; Second, our study is cross-sectional—effectively a comparison of two snapshots (2010 and 2020) rather than a continuous observation of change. This design means we cannot definitively establish causal relationships or the direction of influence over time. A longitudinal or panel study following cities over multiple census periods could help untangle cause and effect, and confirm whether improving physical activity leads to slower aging or if rapidly aging communities respond by becoming more active. Third, our model does not include certain psychosocial and cultural factors that could be important. Aging is not only a function of births, deaths, and behaviors, but also of social context—for instance, attitudes toward family size, elder care traditions, community engagement of seniors, and migration decisions of younger generations. Incorporating such social variables could further enrich the analysis, for example, that communities with strong social support networks age “better” even if physical activity is high or incomes are low. Moreover, city-level physical-activity data were unavailable for 2010, limiting a fully symmetric panel; although our common-subset analysis suggests stable results, future research should employ continuous PA series once released. Future studies should consider health-adjusted aging indices or include variables like life expectancy, disability rates, or quality of life of the older adults to paint a fuller picture of aging dynamics.

Despite these limitations, our study provides a nuanced, multi-scalar understanding of population aging in Hubei. It emphasizes that the drivers of aging are evolving over time and vary across space, and that policy responses must adapt accordingly. Theory-driven analysis combined with practical awareness of local versus regional factors can help policymakers design interventions that promote healthy and balanced aging – ensuring that longer lives are enjoyed in all communities, not just reflected in aggregate statistics.

## 5 Conclusion

Building on our MGWR analysis of population aging in Hubei Province, this study offers four messages:

(1) Physical activity is the foremost local driver of aging in 2020: After controlling for demographics, economic affluence, healthcare capacity and environment, city-level physical activity showed the largest absolute MGWR coefficient, with roughly half of all cities exhibiting statistically significant negative effects. This underscores that sedentary lifestyles now outweigh structural factors in determining which communities age fastest or slowest.
(2) In 2010, per capita GDP and healthcare facility density displayed the highest spatial variability, indicating that economic and service disparities drove aging patterns. By 2020, these disparities had largely converged (SD < 0.10), whereas the dispersion of birth-rate effects surged nine-fold (SD ≈ 0.42), making fertility decline the most uneven demographic process shaping city-level aging.
(3) MGWR delivers richer insights than global or single-bandwidth models: Compared to OLS and classic GWR, MGWR achieved superior fit (Adj.R^2^ = 0.71; AICc = –18.4) and captured variable-specific spatial scales. This multi-scale flexibility exposed the layered nature of aging drivers—revealing, for instance, that a one-size-fits-all bandwidth would have masked the outsized role of activity or flattened fertility heterogeneity.
(4) MGWR reveals that physical activity effects operate at a very local scale, while demographic and economic influences span broader regions. Active-aging interventions should be designed and managed by city authorities, whereas fertility incentives and economic development policies warrant coordination at the provincial or multi-city level.

Our findings highlight the need for a dual strategy: tackle structural demographic pressure by supporting fertility in low-BR hot spots, and simultaneously deploy modifiable behavioral levers by promoting physical activity—now on par with BR as a key determinant of spatial aging disparities.

## Data Availability

The original contributions presented in the study are included in the article/supplementary material, further inquiries can be directed to the corresponding authors.

## Footnotes

^1^ https://github.com/pysal/mgwr
